# Supplementary material for: Dynamics of brain-muscle networks reveal effects of age and somatosensory function on gait
Source: iScience. 2024 Feb 9;27(3):109162. doi: 10.1016/j.isci.2024.109162 (PMC10897916; doi:10.1016/j.isci.2024.109162)
Supplement: Document S1. Figures S1–S8 and Tables S1 and S2 [file mmc1.pdf]

iScience, Volume *27*

Supplemental information

Dynamics of brain-muscle networks reveal effects of age and somatosensory function on gait

Luisa Roeder, Michael Breakspear, Graham K. Kerr, and Tjeerd W. Boonstra

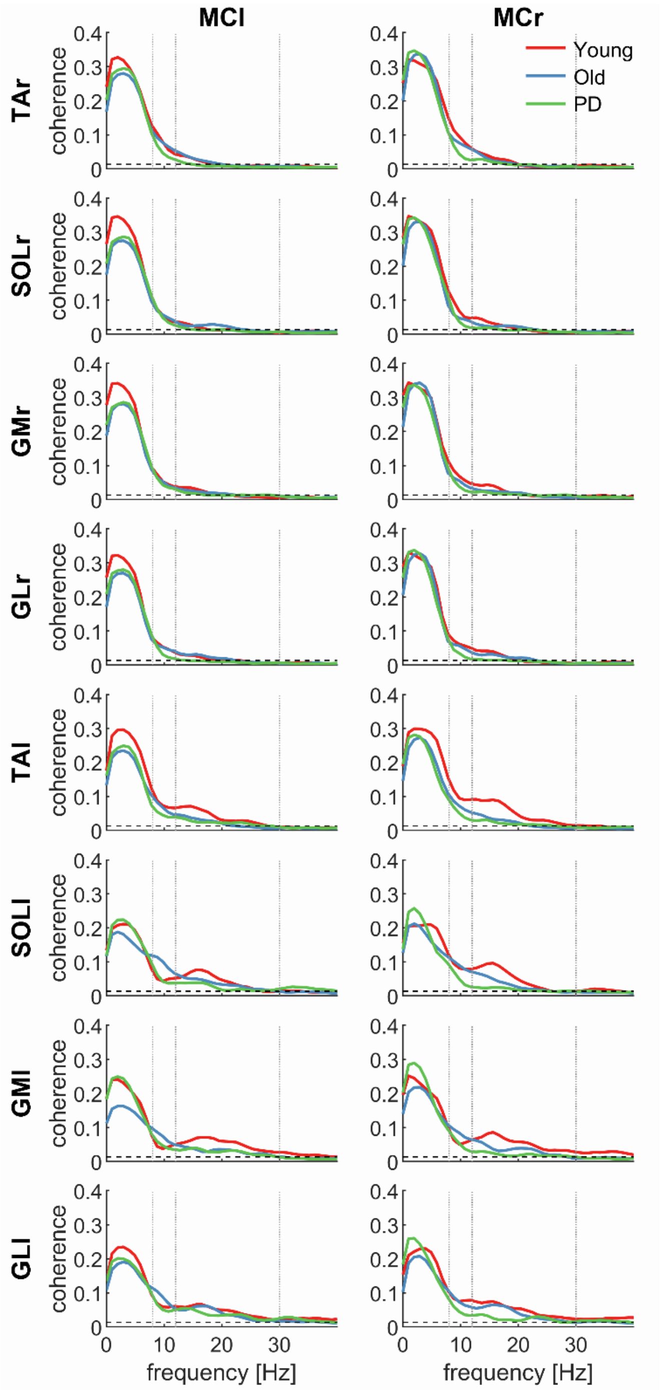

Figure. S1 (related to Figure 1).

Corticomuscular coherence between EEG and EMG channels during double support. Grand-average coherence is averaged over the double support interval and plotted as a function of frequency. The left column shows coherence between the left motor cortex (MCl) and all leg muscles (TA, SOL, GM, GL) on both sides (left indicated with l, right with r). The right column shows coherence between right motor cortex (MCr) and leg muscles. The horizontal dashed lines indicate the the 95% CI (0.014), coherence values above this line are statistically significant. Dotted vertical lines indicate the major frequency bands (alpha at 8-12 Hz, beta at 12-30 Hz). The x-axis shows frequencies in Hz.

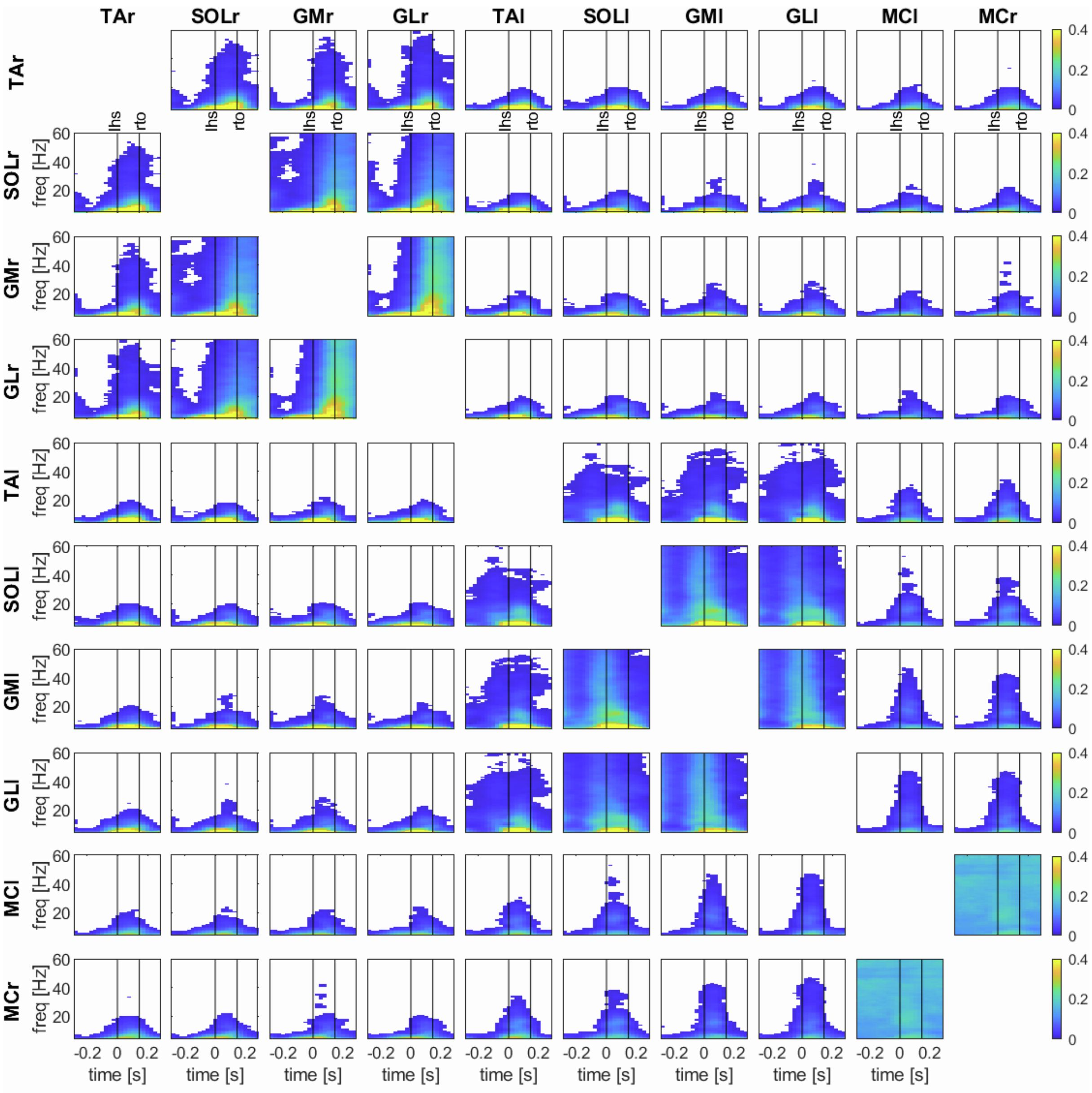

Figure. S2 (related to Figure 1).

Time-frequency coherence between all channel combinations during overground walking in healthy young people. Grand-average coherence is shown between all four leg muscles (TA, SOL, GM, GL) on both sides (left indicated with l, right with r) and bilateral motor cortices (MCl, MCr). Coherence values are thresholded: average coherence values below the 95% CI are set to zero (white). The x-axis shows the time in seconds relative to heel strike (t=0) of the left foot and the y-axis the frequencies in Hz. Black vertical lines indicate the footswitch events. lhs, left heel strike; rto, right toe-off.

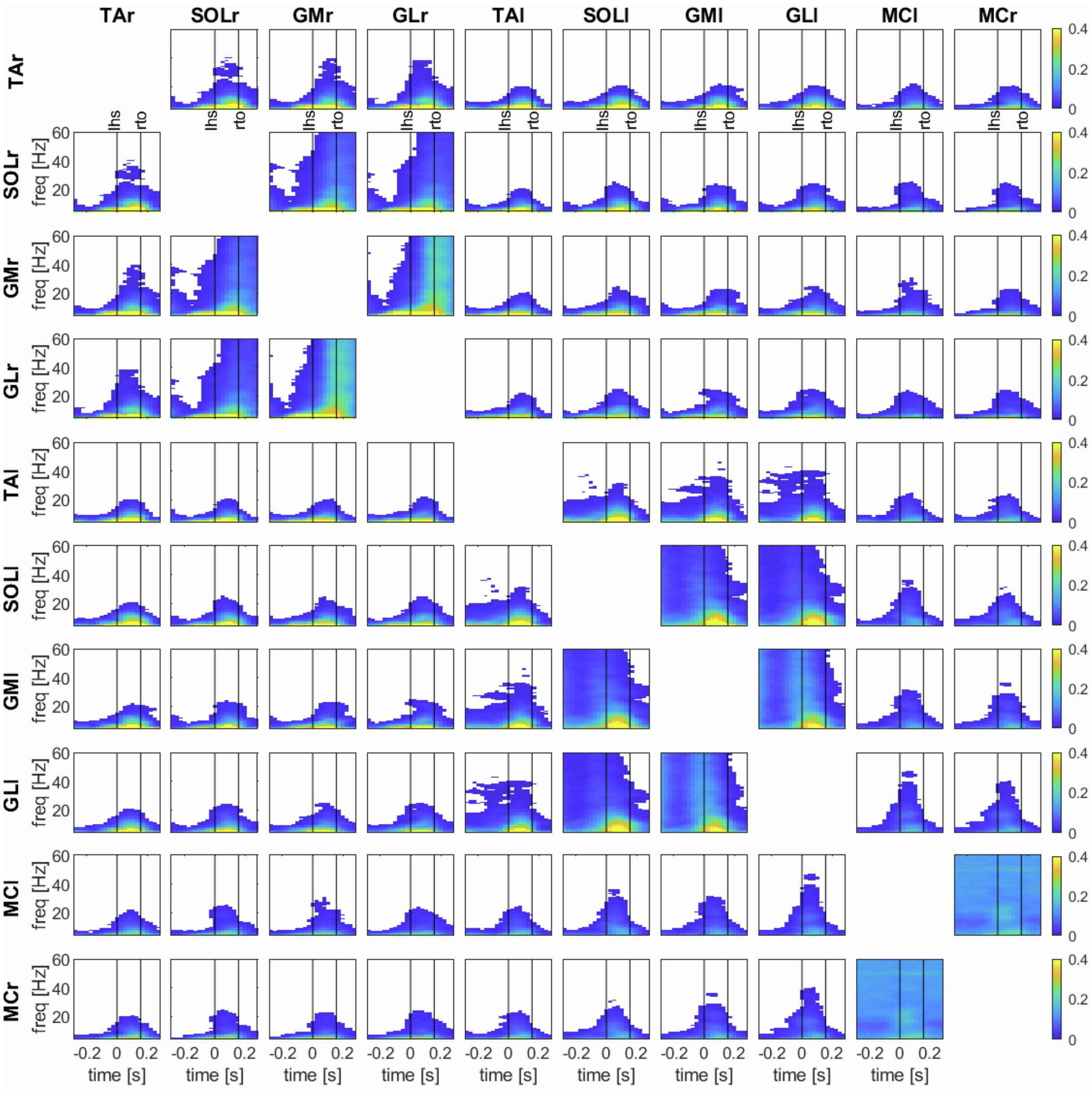

Figure. S3 (related to Figure 1).

Time-frequency coherence between all channel combinations during overground walking in healthy older people. Grand-average coherence is shown between all four leg muscles (TA, SOL, GM, GL) on both sides (left indicated with l, right with r) and bilateral motor cortices (MCl, MCr). Coherence values are thresholded: average coherence values below the 95% CI are set to zero (white). The x-axis shows the time in seconds relative to heel strike (t=0) of the left foot and the y-axis the frequencies in Hz. Black vertical lines indicate the footswitch events. lhs, left heel strike; rto, right toe-off.

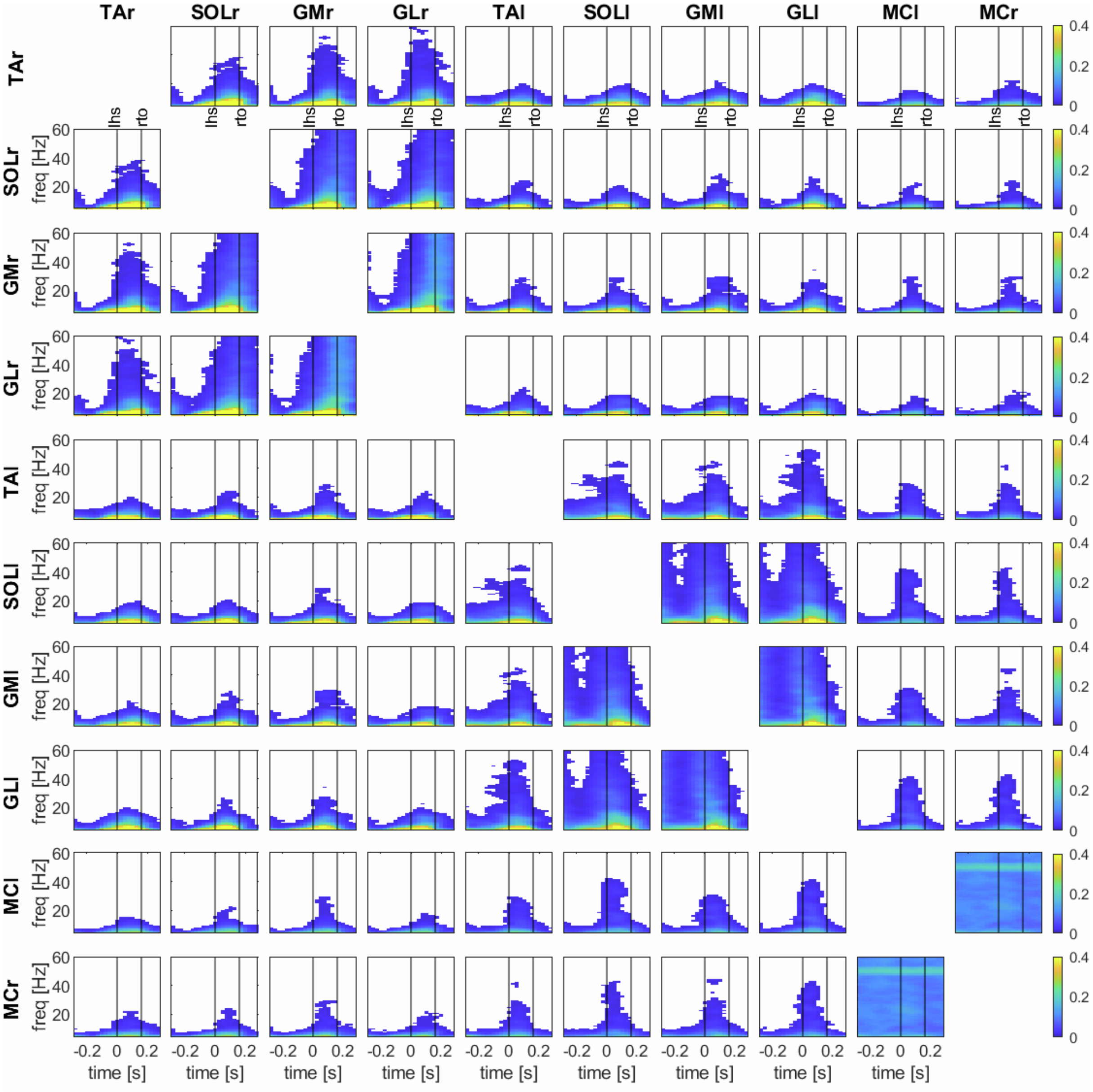

Figure. S4 (related to Figure 1).

Time-frequency coherence between all channel combinations during overground walking in people with PD. Grand-average coherence is shown between all four leg muscles (TA, SOL, GM, GL) on both sides (left indicated with l, right with r) and bilateral motor cortices (MCl, MCr). Coherence values are thresholded: average coherence values below the 95% CI are set to zero (white). The x-axis shows the time in seconds relative to heel strike (t=0) of the left foot and the y-axis the frequencies in Hz. Black vertical lines indicate the footswitch events. lhs, left heel strike; rto, right toe-off.

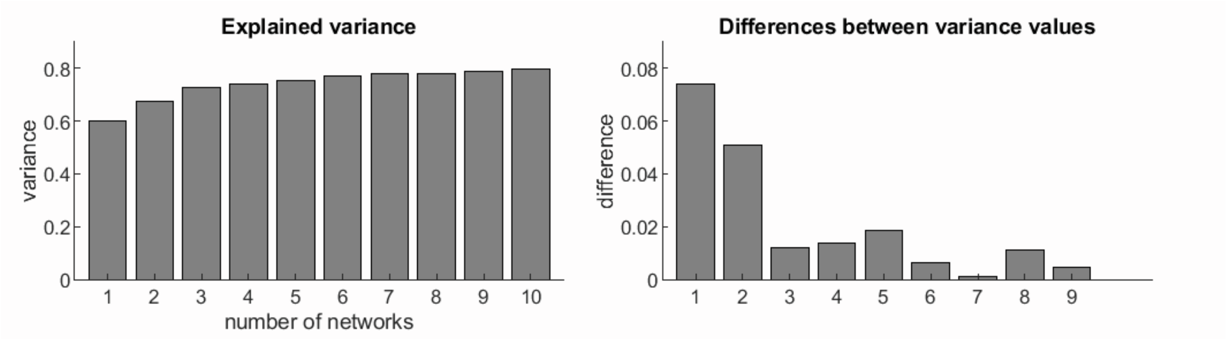

Figure. S5 (related to Figure 2).

Variance explained by components (networks). Cumulative of the variance (left) explained by extracting 1-10 networks using non-negative matrix factorization (NNMF). Relative increase in explained variance when additional component is added (right). The scree plot shows that the additional explained variance levels off after adding two additional networks. Based on this criterion we decided to extract three networks, which together explained 72.7% of the variance.

To extract the low-dimensional subspace in which the brain-muscle networks unfold, we decomposed time-frequency coherence in canonical frequency bands using orthogonal NNMF. Three orthogonal components (networks) were extracted that explained most of the variance (72.7%). We used the scree plot criterion to determine this meaningful number of dimensions in our latent dimension-reduction analyses (Figure S4).

That is, the difference in variance between network 1 and 2 (= 0.074) and between 2 and 3 (= 0.05) was largest; all other networks add very small increments to the explained variance (< 0.02).

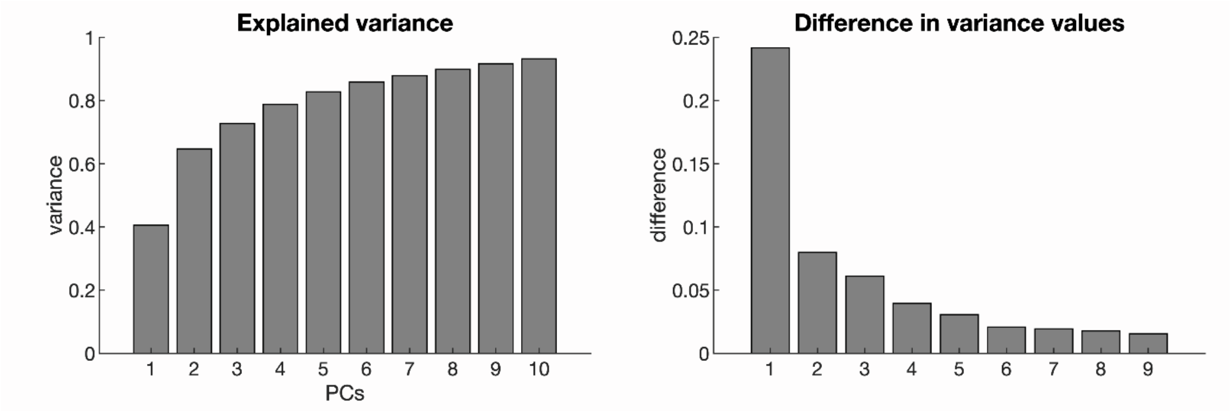

Figure. S6 (related to Figure 5).

Variance explained by principal components. Cumulative of the variance (left) explained by extracting 1-10 principal components (PCs). Relative increase in explained variance when additional component is added (right). The scree plot shows that the additional explained variance levels off after adding one additional PC. Based on this criterion we decided to include two Pcs, which together explained 65% of the variance.

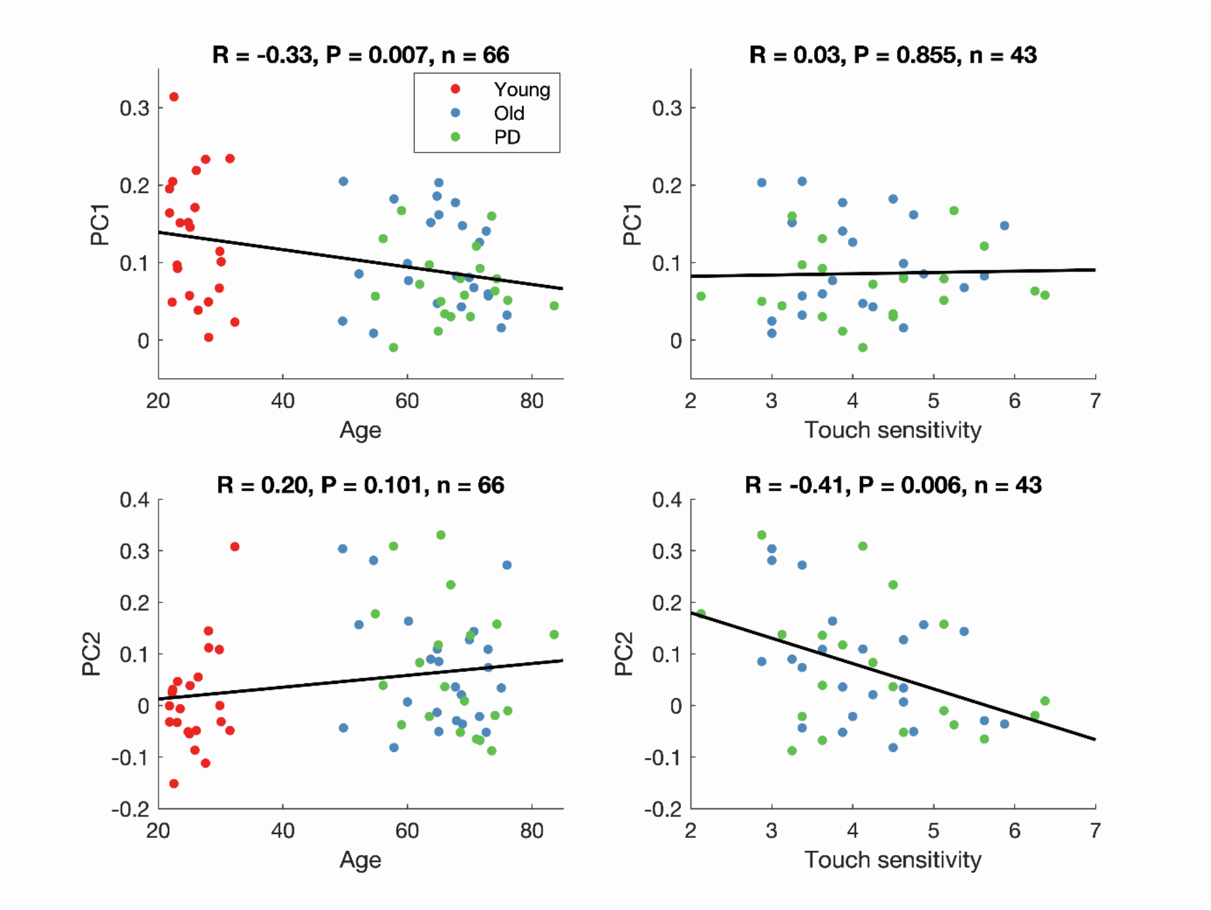

Figure. S7 (related to Figure 5).

Correlation effects of network activations and participant & functional measures. Higher "TouchTotal" scores (touch sensitivity) indicate worse touch sensitivity.

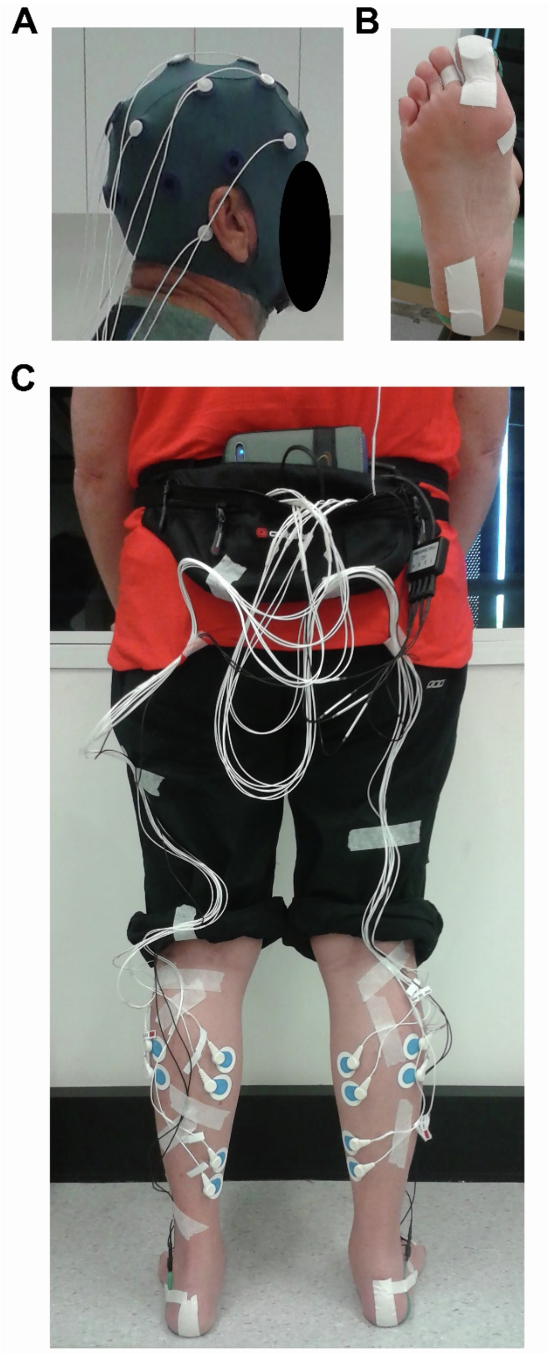

Figure. S8 (related to STAR Methods data acquisition).

Sensors used for data acquisition. EEG (A), foot switches attached to the heel and toe (B), EMG electrodes attached to triceps surae muscles and belt bag with TMSi Mobita amplifier system tied around a participants' waist (C).

	PC1			PC2		
	r	p	p_{ajd}	r	p	p_{adj}
Age	**-0.33**	**0.007**	**0.042**	0.20	0.10	0.30
Sex	0.17	0.16	0.39	-0.28	0.025	0.10
Weight	-0.11	0.38	0.56	-0.12	0.33	0.56
$TouchSensitivity_{total}$	0.03	0.86	0.86	**-0.41**	**0.006**	**0.042**
$UPDRS_{motor}$	-0.23	0.33	0.56	-0.09	0.72	0.85
Hoehn&Yahr	-0.17	0.47	0.62	0.07	0.78	0.85

Table S1 (related to Figure 5).
Correlation between the eigenvector coefficients and participant characteristics and clinical scores.

**Tactile sensitivity**	**Healthy old (n=24)**		**PD (n=20)**		**Group effect**	
	Mean	SD	Mean	SD	t-value	p-value
Ankle	4.02	0.78	4.63	1.23	-1.95	0.059
Toe	3.39	1.11	3.55	0.94	-0.50	0.62
Medial plantar	3.63	1.51	3.83	1.54	-0.42	0.68
Heel	5.41	1.11	5.05	1.70	0.84	0.41
Total	4.11	0.85	4.26	1.12	-0.49	0.63

Table S2 (related to Figure 5).
Tactile sensitivity (threshold 1 to 8) of healthy older and PD groups.
